# Phosphoproteomics Data-Driven Integrative Analysis of Autophosphorylation Sites in the Human Kinome

**DOI:** 10.3390/ijms27156745

**Published:** 2026-07-28

**Authors:** Athira Perunelly Gopalakrishnan, Mahammad Nisar, Alimath Sambreena, Radul R. Dev, Prashant Kumar Modi, Sowmya Soman, Rajesh Raju

**Affiliations:** 1 Centre for Integrative Omics Data Science (CIODS), Yenepoya (Deemed to be University), Mangalore 575018, India; ammuperunelly@gmail.com (A.P.G.);; 2 Center for Systems Biology and Molecular Medicine (CSBMM), Yenepoya Research Centre, Yenepoya (Deemed to be University), Mangalore 575018, India; prashantmodi@yenepoya.edu.in

**Keywords:** kinase, phosphorylation, dark phosphosites, autophosphorylation, mass-spectrometry

## Abstract

Many kinases are known to homo- or hetero-merize and phosphorylate themselves or each other. Phosphorylation by itself (autophosphorylation) is mechanistically perceived as either cis- or trans-interactions. Identification of autophosphorylation sites in kinases provides an opportunity to assess the activity of the kinome in phosphoproteome datasets as an efficient indicator of kinase activity. In order to predict kinase activity-associated autophosphorylation sites, we first encompassed the currently reported homodimeric interactions between kinases from various sources. Subsequently, we accounted for the autophosphorylation sites in kinases that are predicted by phosphomotif screening approaches, in vitro kinase assays, and those perceived based on phosphomotif mutant analysis. Together, we identified 4184 autophosphorylation sites in 386 kinases, and among them, 315 kinases were reported with homodimeric protein–protein interactions. Furthermore, we analysed 361 kinases that harbor multiple autophosphorylation sites and 25 kinases with a single autophosphosite based on contemporary kinase–substrate interactions. Among the 386 kinases, autophosphosites for 17 kinases were derived through high-throughput or low-throughput approaches, and for 80 kinases, they were predicted based on their phosphomotif analysis. The remaining 289 were based on predictions by multiple tools or were reported in more than one dataset. There were many instances in which, although one auto-phosphorylation site was reported, multiple other sites were also predicted. In this regard, we analysed the instances of phosphosites in kinases that are already associated with the induction or inhibition of kinase activity and identified 223 kinases with one or more sites with functional association. To evaluate their potential as auto-phosphorylation sites, we systematically assembled 1572 profiles and 978 differential cellular phosphoproteome datasets. Subsequently, we accounted for the frequency of co-detection or co-differential regulation of these autophosphosites with multiple kinase activity-associated sites within each kinase and further accounted for their known/predicted substrate phosphorylation sites. Together, we provide a reference compendium of autophosphorylation sites secluded in the human kinome and their co-regulation with substrates based on global phosphoproteome datasets, AutoPhosDb. As indicators of kinase activity, these phosphosites in kinases would serve as a surrogate to enrich the active kinases and their potential substrate-specificity in phosphoproteome datasets.

## 1. Introduction

Protein kinases are pivotal regulators of cellular signaling, coordinating the phosphorylation process that governs a wide array of biological processes, including cell proliferation, differentiation, metabolism, and apoptosis. These enzymes act as molecular switches, transferring phosphate groups from ATP to serine, threonine, or tyrosine residues on substrate proteins, thereby modulating their activity, localization, and interaction potential [1,2]. Dysregulation of kinase activity has been implicated in a wide range of diseases, including cancer, neurodegenerative disorders, and autoimmune conditions, etc. [3].

A critical regulatory mechanism within the kinase family is autophosphorylation, where kinases catalyze the phosphorylation of their own residues. This self-modification process plays essential roles in modulating enzymatic activity, promoting structural stabilization, and fine-tuning substrate affinity and specificity [4]. Autophosphorylation can occur in *cis* (intramolecular) or in *trans* (intermolecular) mode, with the latter often requiring dimerization or oligomerization of kinase domains [5]. Key structural and biochemical studies—such as those by Johnson and Lewis (2001) and Zhang et al. (2006)—have demonstrated that autophosphorylation is essential for the activation mechanisms of several kinase families, including receptor tyrosine kinases and cyclin-dependent kinases [4,6].

Recent studies on phosphorylation events have deepened our understanding of how kinases achieve self-phosphorylation or autophosphorylation. Beenstock et al. (2016) described how certain kinases adopt “prone-to-autophosphorylate” conformations that facilitate self-modification even before full activation, emphasizing the structural flexibility and regulatory sophistication of this process [7]. Similarly, structural analysis of fibroblast growth factor receptor 1 (FGFR1) revealed that its autophosphorylation proceeds through a sequential and ordered mechanism that enables precise temporal control of activation [8]. Studies of p21-activated kinase 1 (PAK1) further showed that distinct autophosphorylation events within both regulatory and catalytic domains differentially control kinase activity and effector binding [9]. Comprehensively, these findings highlight autophosphorylation as a versatile regulatory switch embedded across the kinome.

Importantly, dysregulation or mutation of autophosphorylation sites has emerged as a critical determinant in human disease. Variants that abolish, mimic, or introduce novel autophosphorylation events can perturb kinase activation dynamics, substrate recognition, or localization, resulting in pathological signaling states. For instance, mutations in the PTEN-Induced Kinase 1 (PINK1), which alter its autophosphorylation pattern, compromise Parkin recruitment and mitophagy, contributing to familial Parkinson’s disease [10,11]. Similarly, ABL Proto-Oncogene 1 (ABL1) activation-loop mutations affecting Tyr253 are recurrent in leukaemia and disrupt the finely tuned balance between autoactivation and substrate phosphorylation [12]. In receptor kinases such as MET Proto-Oncogene (MET) and Discoidin Domain Receptor Tyrosine Kinase 2 (DDR2), pathogenic substitutions at autophosphorylation tyrosines (e.g., Y1234 and Y740, respectively) lead to constitutive activation and are associated with congenital malformations and oncogenesis. Moreover, variants in Calcium/Calmodulin Dependent Protein Kinase II Alpha (CAMK2A) (Thr286) and RPS6KA3 (Ser227) are linked to neurodevelopmental disorders such as intellectual disability and Coffin–Lowry syndrome, respectively, underscoring the broad clinical relevance of these regulatory hotspots [13,14].

Comprehensive analysis of our autophosphorylation sites curated in this study with ClinVar [15] and OMIM [16] annotations revealed that several functionally important autophosphosites coincide with reported pathogenic variants. These include mutations in CAMK2A (T286, RCV00051), MET (Y1234, RCV00062), LYN Proto-Oncogene (LYN) (Y508, RCV00251), ABL1 (Y253, RCV00001), Transforming Growth Factor Beta Receptor 2 (TGFBR2) (Y336, RCV00001), DDR2 (Y740, RCV00075), Ribosomal Protein S6 Kinase A3 (RPS6KA3) (S227, RCV00072), and Transient Receptor Potential Cation Channel Subfamily M Member 7 (TRPM7) (T1482, RCV00000). Such colocalization between autophosphorylation residues and pathogenic variants suggests that perturbation of self-regulatory phosphorylation is a unifying mechanism in kinase-linked pathologies. These associations emphasize the importance of integrating biochemical, structural, and clinical data to interpret kinase variant pathogenicity and to identify autophosphorylation sites as potential therapeutic targets.

In this study, we present an integrative framework for identifying kinase activity-associated signature autophosphorylation sites. By combining mass spectrometry-based phosphoproteomic studies, motif-based PSPA analyses, and multi-layered datasets, including inhibitor perturbation and clinical variant annotation, we overcome key limitations of traditional experimental approaches. Our analysis reveals novel, potentially functional autophosphorylation residues associated with kinase activation, many of which remain uncharacterized yet exhibit regulatory potential based on co-regulation with known activation sites or inhibitor sensitivity. In summary, these findings provide a resource for the mechanistic dissection of kinase regulation and offer new opportunities for linking phosphosite dysregulation to disease mechanisms and therapeutic intervention.

## 2. Results

**Figure 1:**
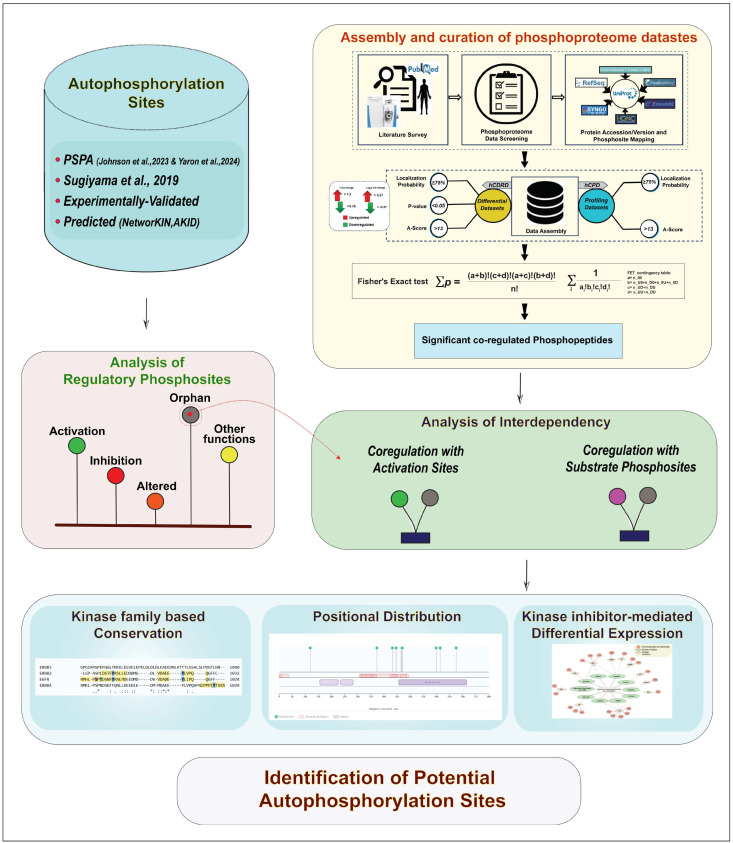
Analysis of autophosphorylation sites. Workflow for the identification of potential autophosphorylation sites based on global phosphoproteomics datasets [21,22,23].

Identification of phosphosites in kinases with autophosphorylation potential is essential to evaluate their activation status or specificity towards substrates. Many of the experimentally validated and predicted sites are also considered sites of phosphorylation by other kinases. In this regard, a better understanding of the autophosphorylation potential of these sites can contribute to enhanced analysis of kinase–substrate interactions in global or phosphomotif-enriched phosphoproteome datasets. Therefore, this study provides a first compendium of potential autophosphorylation sites detected in kinases. The overview is given in the workflow (Figure 1).

### 2.1. Human Kinome Homomers

Many kinases are known to form homomers or heteromers to regulate the kinase activity of each other or mediate cellular functions in synergy. Homomerization is also considered one of the major modes of induction of their kinase activity, potentially through autophosphorylation [17,18,19,20]. Notably, possibilities of both the cis- and trans-autophosphorylation are also perceived, and hence, homodimerization may not be essential for all kinases. To visualize the potential for such interactions, we first assembled the homodimer interactions of kinases. Based on the reported studies, currently, 315 kinases have been reported to form homomers, including dimers or tetramers (Table S1).

### 2.2. A Landscape of Autophosphorylation Sites in the Human Kinome

**Figure 2:**
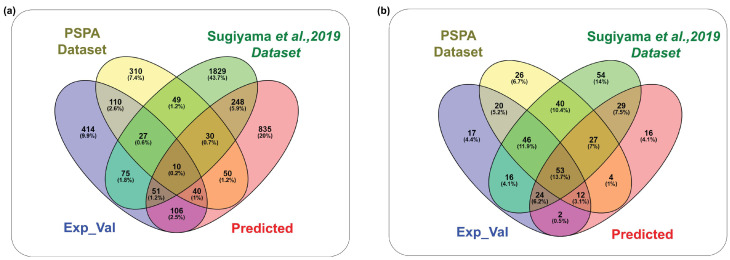
Distribution of autophosphorylation sites. (**a**) Number of autophosphorylation sites from different datasets; (**b**) number of kinases associated with autophosphorylation from different datasets [21].

There were 4184 phosphosites in 386 kinases that are currently considered autophosphorylation sites based on various approaches, including high-throughput and low-throughput experiments, as well as predictions by different tools such as AKID and NetworKIN (Figure 2). Moreover, phosphomotif sequence variant-based screening approaches have led to 359 potential sites that have not yet been evaluated using in vitro or in vivo experimental approaches.

**Figure 3:**
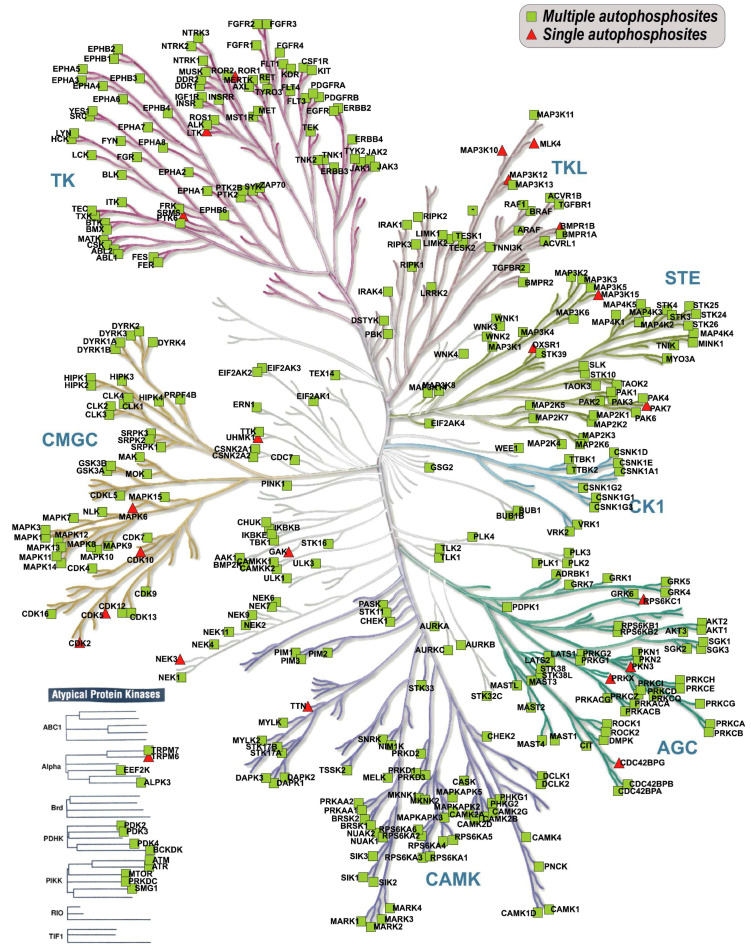
Kinome-wise distribution of autophosphorylation sites. Kinome tree represented the partition of single and multiple autophosphorylation sites predicted in each kinase.

In total, 386 kinases possess at least one autophosphorylation site. Among them, 20 kinases are currently reported to harbor one auto-phosphorylation site (Figure 3). Interestingly, multiple phosphosites in a kinase are also validated or predicted with autophosphorylation potential. For example, 56 phosphosites in LRRK2 or 51 phosphosites in TRPM7 kinase are perceivable as their autophosphosites (Table S2). Additionally, the sequence variations and diseases associated with these autophosphorylation sites are provided in Table S3.

### 2.3. Exploration and Assembly of Global Phosphoproteome Datasets

To analyse the dependencies between autophosphosites and other phosphosites in various phosphoproteomics experimental conditions, we have curated phosphoproteomics datasets from the literature. We conducted a systematic literature search on PubMed using the query “phosphoproteome” OR “phosphoproteomics” NOT “Review” NOT “plant”, which resulted in a total of 3567 phosphoproteomics studies. From these studies, we have filtered mass-spectrometry-derived human-cell-line-specific conditions. From 303 studies, we assembled 1572 profiling datasets and 978 differential datasets.

Class I phosphosites or phosphosites with a localization probability of ≥75% or an A-score of >13 were considered in the further analysis of profiling datasets. Similarly, phosphosites with a localization probability of ≥75% or an A-score > 13 and a *p*-value < 0.05 were considered in differential datasets. Additionally, a fold change cutoff of 1.3 was arbitrarily chosen to classify differentially regulated, upregulated, and downregulated phosphosites from the differential datasets [24,25,26].

**Figure 4:**
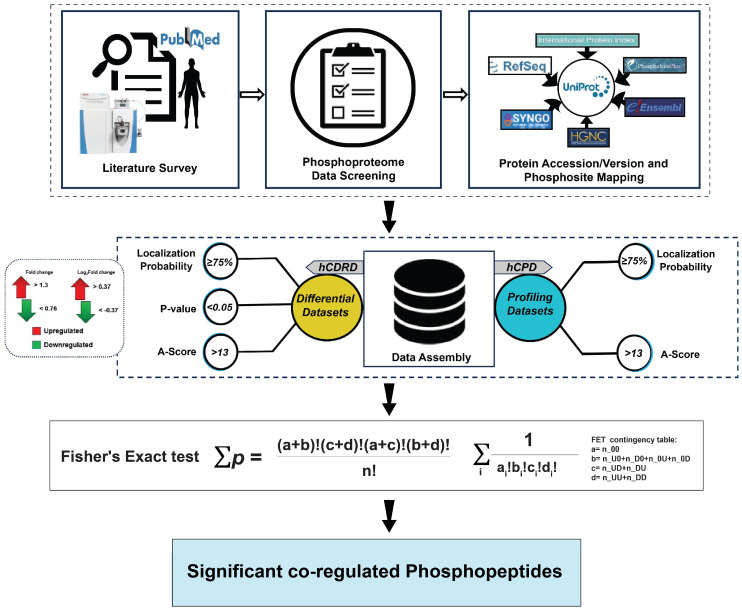
Data curation and assembly of global phosphoproteomics datasets. Workflow for phosphoproteomics data curation and assembly. hCDRD: Human Cellular Differential Datasets; hCPD: Human Cellular Profiling Datasets.

Profiling and differential datasets included Class I phosphosites identified under different experimental conditions, such as drug treatment, gene knockdown, growth factor stimulation, or mutation experiments, etc. These datasets provide a qualitative assessment of phosphosite detectability across various biological contexts. Each experimental condition, including individual time points in temporal studies, was treated as a distinct profiling dataset. Differential datasets, in contrast, focused on relative quantitative phosphopeptide expression levels between test and control conditions. Differential regulation was determined based on the fold change (FC) threshold: phosphopeptides with an FC ≥ 1.3 were considered upregulated, while those with an FC ≤ 0.76 were considered downregulated. The workflow for the curation and assembly of the phosphoproteomics datasets is given in Figure 4.

### 2.4. Evaluation of Autophosphorylation Sites in the Human Kinome Using Phosphoproteome Datasets

**Figure 5:**
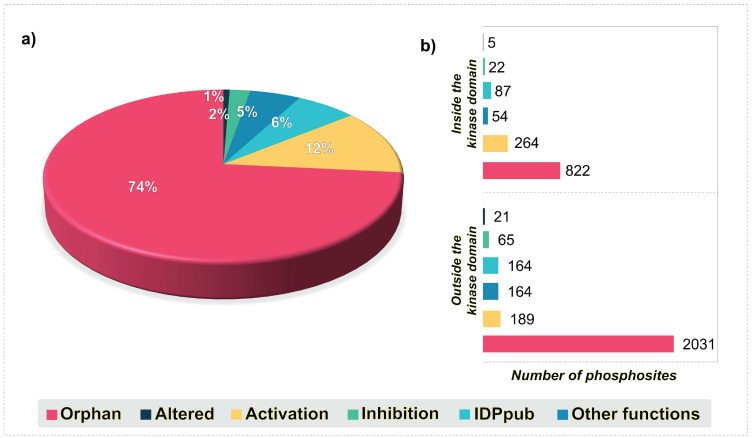
Regulatory autophosphorylation sites. (**a**) Distribution of regulatory functions among the autophosphorylation sites. (**b**) Protein kinase-based distribution.

Functional classification of autophosphorylation sites identified 516 activation sites, which correlated with increased kinase activity, and 88 inhibition sites, which served as negative regulatory elements. Among the remaining phosphorylation sites, 3335 lacked regulatory function annotations in PhosphoSitePlus; however, literature evidence for 262 of these sites was retrieved from IDPpub. Additionally, 3073 orphan sites lacked functional annotation in existing databases, prompting further analysis (Figure 5a). By integrating phosphoproteomics datasets, we evaluated the frequency of co-detection and co-differential regulation of these orphan sites with known substrates and known regulatory phosphosites for each kinase. Based on their shared regulatory patterns, we assigned functional relevance to potential orphan phosphosites, suggesting roles in kinase activation and downstream signaling.

We also investigated the domain-level distribution of autophosphorylation events across the kinome. Of the 4184 autophosphorylation sites, 1733 mapped to annotated functional domains. Strikingly, 1254 of these phosphosites resided within the core protein kinase domain, yet most remain functionally unannotated, representing a substantial set of orphan regulatory sites (Figure 5b).

Additionally, we have analyzed the distribution of these phosphorylation sites across the disordered region in the kinome. From the analysis of the distribution of autophosphorylation sites across the disordered regions in the protein structure, we have identified that among the 4184 autophosphorylation sites, 1113 sites were situated inside the disordered region, and 2269 sites were outside the disordered region (Table S2).

### 2.5. Interdependency Between Known Regulatory Sites Within Kinases

Positive coregulation of an orphan site with either activation or inhibition sites may suggest a functional link to kinase regulation. When an orphan site is positively coregulated with known activation or inhibition sites, this may reflect a shared regulatory role in kinase signaling. In contrast, negative coregulation offers potential clues to opposing functions. A negative correlation between an orphan site and an inhibition site may suggest that the orphan site contributes to kinase activation. Alternatively, a negative correlation with an activation site could indicate that the orphan site plays an inhibitory role.

To explore these hypotheses, we analyzed differential phosphoproteome datasets to investigate the potential interdependence of expression between orphan autophosphorylation sites and established activation sites within the same kinases (Table S4). Notably, within MTOR, the activation site S2481 exhibited strong co-regulation with autophosphorylation site S2478. Similarly, in EPHA2, the activation site, Y594, was associated with the autophosphorylation site, Y575. In RIPK2, the activation site S176 showed a strong positive correlation with the autophosphorylation site S178. Also, activation site S380 in RPS6KA1 coregulates with the identified orphan autophosphorylation sites S45, S732, and S221 in RPS6KA1 (Table 1A). Conversely, in PRKAA1, a negative co-regulation was observed between the activation site (T183) and the inhibitory site (S496), both of which showed significant anti-correlation with the validated autophosphorylation site (S360). Additionally, inhibitory phosphorylation site S496 in PRKAA1 negatively coregulated with S356 and T355, other than S360 (Table 1B). These observations suggest that many orphan sites can be classified into autophosphorylation sites that may contribute to the functional regulation of their respective kinases.

### 2.6. Analysis of Potential Activity-Associated Autophosphorylation Sites Based on Co-Regulation with Known Substrate Phosphosites

The interdependency between a phosphosite and its downstream substrates holds significant functional relevance, as it can reveal potential regulatory roles within kinase signaling pathways. When the phosphorylation of a specific site, particularly an orphan site, consistently correlates with the phosphorylation of known downstream substrates, it suggests a mechanistic connection between the two events. This interdependence implies that the site may influence or even enable substrate phosphorylation, either through kinase activation, allosteric modulation, or scaffolding functions. In the context of autophosphorylation, such co-occurrence patterns can point to a regulatory autophosphosite that governs the kinase’s ability to transmit signals downstream. Unlike sequence motifs, which offer static predictions, interdependency captures dynamic, condition-specific relationships that reflect real-time signaling events. Therefore, evaluating these dependencies provides a powerful strategy to infer their function, prioritize phosphosites for experimental validation, and improve our understanding of the contextual regulation of cellular signaling networks.

To analyze the potential autophosphorylation sites associated with kinase activity, we explored the interdependency between orphan autophosphosites and their experimentally validated or phosphomotif-enriched downstream substrates. For each kinase, based on the shared frequencies between autophosphosites with known substrates, we ranked each orphan site. The phosphosites with higher shared frequencies in positively and negatively coregulated datasets, with a ratio ≥ 25, were considered potential autophosphorylation sites.

From the differential data, we have identified 60 orphan potential auto-phosphorylation sites positively coregulated with the downstream substrate phosphosites with a ratio of ≥25 (Table S5). Highly positively coregulated sites (Ratio ≥ 40) are given in Table 2. Potential coregulating phosphosite pairs were in accordance with the defined significance thresholds of Fisher’s exact test (FET) with *p* < 0.05 and *q* < 0.05.

### 2.7. Analysis of Potential Activity-Associated Autophosphorylation Sites from Profiling Datasets

In parallel with the differential dataset analysis, we examined the co-detection patterns of orphan phosphosites together with their respective substrate phosphorylation sites. This analysis revealed a subset of sites that consistently exhibited high co-detection across numerous experimental conditions. The most significant co-detected pairs, defined by a co-detection frequency exceeding 600, are summarized in Table 3. From these data, we can infer that, based on profiling experimental conditions, the orphan autophosphorylation sites STK10_S438, RPS6KA3_S715, and AAK1_T606 may play a role in regulating kinase activity. The other co-detected pairs were listed in Table S6.

**Figure 6:**
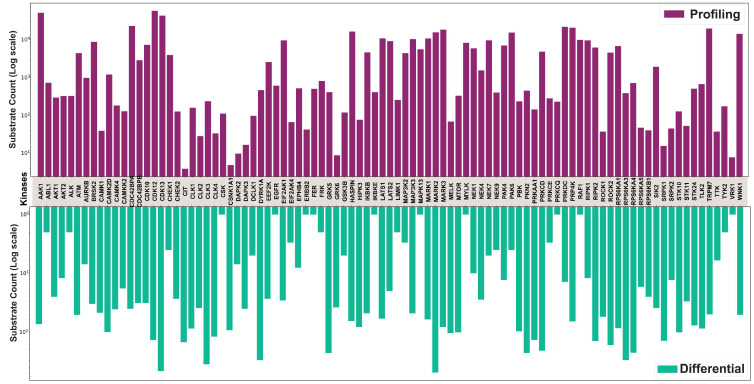
Dataset-based comparison of interdependency. Comparative representation of kinase–substrate co-detection/regulation counts in profiling and differential datasets.

To evaluate the co-regulation of substrate phosphorylation sites with their corresponding orphan autophosphorylation sites, substrate counts identified in both profiling and differential datasets are depicted in Figure 6. This figure depicts a comparative representation of kinase–substrate co-detection counts, with the *x*-axis showing kinases and the *y*-axis showing the number of substrates detected. The profiling dataset (upper, purple) reflects baseline phosphorylation patterns, whereas the differential dataset (lower, green) captures condition-specific changes. Kinases with consistently high counts across both datasets, such as CDKs and MAPKs, are indicative of central signaling hubs, while enrichment in only one dataset distinguishes between qualitative (profiling) and quantitative (context-dependent) (differential) activities.

### 2.8. Kinase Inhibition Reveals Regulatory Expression Patterns of Activity-Associated Autophosphosites

**Figure 7:**
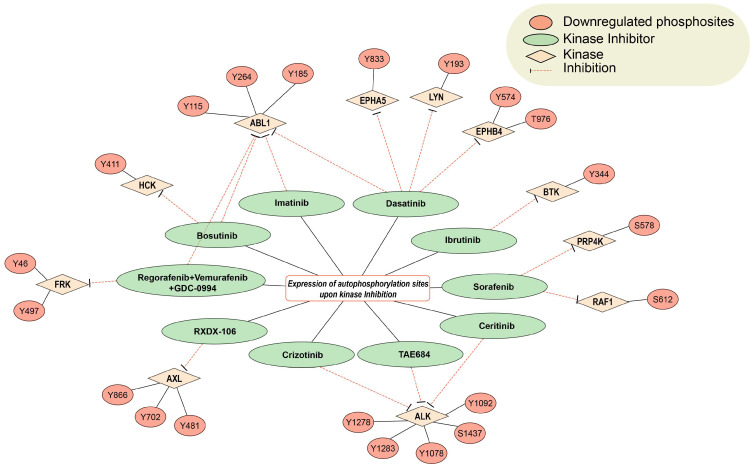
Kinase inhibition and orphan autophosphorylation sites. Potential autophosphorylation sites downregulated upon kinase inhibition.

Kinase inhibition revealed distinct regulatory expression patterns of activity-associated autophosphosites. We curated 29 kinase inhibition-specific differential datasets from nine published studies, encompassing experimental contexts involving inhibition of kinases such as Anaplastic Lymphoma Receptor Tyrosine Kinase (ALK), ABL Proto-Oncogene 1 (ABL1), ephrin type-A receptor 5 (EPHA5), ephrin type-B receptor 4 (EPHB4), tyrosine-protein kinase Lyn (LYN), tyrosine-protein kinase BTK (BTK), tyrosine-protein kinase FRK (FRK), serine/threonine-protein kinase PRP4 homolog (PRP4K), RAF proto-oncogene serine/threonine-protein kinase (RAF1), tyrosine-protein kinase HCK (HCK), and tyrosine-protein kinase receptor UFO (AXL), among others (Figure 7, Table S7). Within these datasets, we identified 21 orphan autophosphorylation sites across 11 kinases that were consistently downregulated upon inhibition of their corresponding kinases. As expected, activation-associated sites typically decreased in response to inhibitor treatment, and these observations suggest that the identified autophosphosites may play functional roles in regulating kinase activity.

### 2.9. Kinase Family-Based Conservation Analysis of Autophosphosites and Structural Mapping

Extending our previous work (Gopalakrishnan et al.), we systematically evaluated the conservation of autophosphorylation sites across the kinase family [27]. A quantitative conservation score (ranging from 0 to 1) was derived from sequence alignment-based features, including sequence similarity, physicochemical property matching, gap penalties, and residue propensity. Phosphorylation sites scoring ≥ 0.75 were defined as highly conserved, whereas those with scores ≤ 0.25 were categorized as poorly conserved. Conservation scores for each phosphosite based on kinase family and interpretation can be accessed from the database “AutoPhosDb”.

To further contextualize our findings, we examined the positional orientation of orphan autophosphorylation sites with respect to key structural regions, including the kinase domain and activation loop. Representative examples highlighting enrichment near the protein kinase domain are provided (Figure S1, Table S8).

### 2.10. AutoPhosDb

Integrating all results and analyses from the present study, we established a resource termed AutoPhosDb. This database facilitates the analysis of autophosphorylation sites across the human kinome, encompassing their positional distribution with respect to kinase domains and intrinsically disordered regions, along with their regulatory roles and kinase family-based conservation. The database is available at: https://ciods.in/autophosdb.

## 3. Discussion

This study explores and presents a resource of kinase activity-associated autophosphorylation sites, many of which remain functionally uncharacterized. The findings from this approach open avenues to elucidate new regulatory mechanisms and signaling roles across different kinases. By integrating mass spectrometry-based identification with phosphomotif-driven approaches, this study addresses limitations inherent to traditional experimental methods, enabling the identification of both known and novel autophosphorylation sites with higher confidence.

Our framework integrates multi-layered datasets, including phosphoproteomics data, motif analysis, and kinase inhibitor perturbations, to reduce false positives and strengthen biological interpretation. Such an integrative approach facilitates the detection of context-dependent phosphorylation dynamics that may otherwise be overlooked by conventional single-method strategies. The inclusion of kinase inhibitor data provides a functional dimension, linking site-specific phosphorylation events to kinase activity profiles and cellular signaling states. This data-driven integration enhances the discovery potential for autophosphorylation events that may serve as functional regulators.

Many of the autophosphorylation sites identified herein remain “orphan”; they lack prior annotation or functional characterization. Nevertheless, their co-regulation with canonical activation sites and sensitivity to pharmacological inhibition strongly suggest potential regulatory importance. These features align with previously reported mechanisms where autophosphorylation either activates, stabilizes, or modulates kinase function [8,13,28,29]. By prioritizing such orphan sites, our approach lays the groundwork for targeted biochemical validation and bridges large-scale phosphoproteomics approaches with focused experimental investigations [27].

In alignment with our prior findings, specific phosphosites in AXL (Y779, Y866) and PRKCD (S302, S304) previously reported as functionally relevant [30,31] were again identified as potential orphan autophosphorylation sites in this analysis. This convergence validates our computational predictions and supports the functional significance of these phosphosites, reinforcing the reliability of our integrative framework.

Prior studies have largely relied on individual kinase or kinase-family-specific analysis, which, while robust, are restricted in scale and scope [9]. For instance, Furdui et al. (2006) demonstrated hierarchical autophosphorylation in FGFR1, where phosphorylation of Y653 precedes Y654 to sequentially activate the kinase [8]. Similarly, the sequential phosphorylation mechanism has been confirmed for other kinases such as DYRK1A [29] and ERN1 [32]. However, these approaches typically lack cross-kinase comparability. Our computational and data-driven strategy provides a broader comparative view across multiple kinases and conditions, offering a scalable alternative for systematically mapping autophosphorylation events.

Recent studies have further advanced understanding of autophosphorylation as a regulatory mechanism. For example, Reinhardt & Leonard (2023) provided an extensive review of activation-loop autophosphorylation across kinases, emphasizing its role in structural activation [13]. Wang et al. (2019) identified Tyr198 as the critical autophosphorylation site regulating STK16’s localization and activity [33], while Pyr Dit Ruys et al. (2012) mapped multiple autophosphorylation sites in eEF2K that modulate its enzyme activity [28]. Similarly, Chan et al. (2002) established that autophosphorylation of DNA-PKcs at Thr2609 is essential for DNA double-strand break repair [12]. In contrast, Layton et al. (2012) reported that Ser608 autophosphorylation in the PI3K p85 subunit had no measurable effect on activity [34], underscoring that not all autophosphorylation events are kinase-activity-regulatory and emphasizing the need for targeted experimental validation.

Recent mechanistic work has continued to uncover novel autophosphorylation phenomena. IKK2 was recently shown to undergo dual-specific autophosphorylation, with a non-canonical tyrosine (Y169) playing a role in substrate phosphorylation [35]. Similarly, PINK1 autophosphorylation at Ser465 [36] and massive autophosphorylation of TRPM7’s Ser/Thr-rich domain [37] demonstrate the diverse structural and regulatory consequences of these modifications.

Although our data-oriented bioinformatics approach prioritises activity-associated signature autophosphorylation sites, functional and structural validation remains crucial. Specific approaches such as molecular dynamics simulations, cryo-EM conformational mapping, and site-specific mutagenesis can elucidate whether candidate sites can impact kinase activity, substrate affinity, or protein–protein interactions. Such studies may also clarify whether autophosphorylation occurs in cis or trans (homo- or heterodimeric), as shown in FGFR1 [8] and DYRK1A [29]. Moreover, integrating structural modelling with dynamic simulation will refine the prioritization of autophosphorylation sites attributed to site accessibility and conformational dynamics.

Collectively, our integrative analysis represents a significant step forward in systematically identifying and contextualising kinase activity-associated autophosphorylation sites based on phosphoproteomics studies. By coupling phosphoproteomics evidence with kinase activity profiles and pharmacological perturbation data, this study establishes a foundational reference for future research on autophosphorylation sites and kinase activity regulation mechanisms. The findings not only expand our understanding of kinase regulation but also pave the way for phosphosite-directed therapeutic exploration targeting autophosphorylation-dependent kinase activation mechanisms.

## 4. Materials and Methods

### 4.1. Identification of Autophosphorylation Sites from Public Data Resources

Autophosphorylation sites in human kinases were extracted from multiple resources where datasets were derived based on high-throughput and low-throughput studies/datasets, as well as phosphomotif-based approaches. Phosphomotif-based approaches were curated from published phosphorylation motif-based studies, including Sugiyama et al. [21], and PSPA-oriented studies, including Johnson et al. [22] and Yaron et al. [23]. Additionally, we extracted phosphorylation data from publicly available databases, including PhosphoSitePlus [38] and RegPhos [39], which catalogues experimentally validated phosphosites. Predicted auto-phosphosites were also retrieved from various kinase substrate prediction tools, such as NetworKIN [40] and AKID [41].

### 4.2. Extraction of Human Kinase Homodimeric Complexes Through Integrative Data Analysis

To investigate the relationship between autophosphorylation and kinase dimerization, we extracted kinase–kinase interactions from interactome databases such as BioGRID [42], ConsensusPathDB [43], and RegPhos [39]. This allowed us to identify 315 kinase–kinase interactions that could facilitate cis- or trans-autophosphorylation mechanisms. Mapped these interactions with corresponding phosphosites and analysed autophosphorylation sites among them. Homodimers are separated based on the interactions. Each identified kinase was cross-referenced with experimentally validated auto-phosphosites and predicted sites to assess their auto-phosphorylation potential.

### 4.3. Analysis of the Regulatory Roles of Kinase Autophosphorylation Sites

Regulatory functions associated with each of the autophosphorylation sites were retrieved from the PhosphoSitePlus database [38]. Phosphosites were categorized based on their functional roles: as phosphosites associated with the induction of kinase activity, as “Activation sites”; inhibition of kinase activity as “Inhibition sites”; and those associated with other functions, such as molecular association, intracellular localization, protein stabilization, etc., as “Other functions”. In instances where phosphorylation site information was unavailable in PhosphoSitePlus, literature-derived evidence was retrieved from IDPpub [44]. The remaining phosphosites with no functions or dark phosphosites reported were considered as “Orphan sites”.

In our previous study, we demonstrated the functional relevance of domain-based phosphosite distribution [27]. Accordingly, in the present study, we also examined the domain-level distribution of autophosphorylation sites using domain annotations provided by the InterPro database [45].

### 4.4. Curation and Assembly of Mass Spectrometry-Derived Human Phosphoproteomics Datasets

To analyze autophosphorylation sites and their potential functional dependencies with other phosphorylation events, we curated phosphoproteomic datasets derived from mass spectrometry experiments based on the published literature. We conducted a systematic literature search on PubMed using the query “phosphoproteome” OR “phosphoproteomics” NOT “Review” NOT “plant” to collect phosphoproteomics studies. From the articles, we have screened experiments involving human cell line samples. These studies spanned a variety of biological contexts, including cell proliferation, infection, signaling pathways, and other functional conditions. Datasets were classified into two categories based on the experimental design of each study: profiling datasets and differential datasets. This classification captured a diverse range of experimental setups and sample types. High-confidence phosphosites (Class I) were retained based on specific criteria: localization probability ≥ 75% or A-score > 13, along with a *p*-value < 0.05. Differentially regulated phosphosites were considered with a fold change cutoff of 1.3.

Protein accessions and phosphosites in each dataset were mapped to the latest accessions and phosphosite positions using our in-house Python scripts, accessing the latest versions of UniProt and HGNC databases as reference [46,47]. Additionally, we curated kinase inhibitor-specific differential phosphoproteomic datasets reported in the literature to analyze the regulatory behaviour of autophosphorylation sites following targeted kinase inhibition.

### 4.5. Analysis of the Interdependency of Auto-Phosphorylation Sites on Kinase Activity

We have identified the potential autophosphorylation sites based on the association with kinase activity and analysed the shared frequencies between orphan autophosphorylation sites derived through experimentally validated and PSPA analyses, with the activation/inhibition sites identified within the same kinases. Based on the ratio between positive and negative coregulation frequencies, we identified potential kinase activity-associated orphan autophosphorylation sites.

Additionally, we have analysed the dependencies between the orphan autophosphorylation site(s) and the known substrate(s) of specific kinases. Based on the shared frequencies between them, we identified potential autophosphorylation sites associated with activity from phosphoproteomics datasets in both profiling and differential datasets, which were considered for the analysis.

In addition, we examined how orphan autophosphorylation sites are spatially positioned relative to the kinase domain. We also investigated whether these autophosphorylation sites occur within intrinsically disordered regions based on disorder annotations from the MobiDB database [48].

### 4.6. Sequence Variations in Autophosphorylation Sites and Disease Associations

To investigate the disease associations of autophosphorylation sites, we conducted a comprehensive analysis that integrated known genetic variants with post-translational modification data. We then extracted information from OMIM (Online Mendelian Inheritance in Man), ClinVar, and the Genome Aggregation Database (gnomAD) databases to identify mutations that overlap with these autophosphorylation sites (OMIM [49], ClinVar databases [50], and gnomAD [51,52]). Particular attention was given to pathogenic and likely pathogenic variants, as well as variants of uncertain significance that lie directly at the phosphosite residue.

In addition to direct overlap, we investigated the local sequence context by analyzing the ±7 amino acid window surrounding each autophosphorylation site, referred to as the phosphomotif. This region was chosen to capture proximal residues that may influence kinase recognition, structural conformation, or post-translational regulation. Within these flanking regions, we catalogued all documented missense mutations and assessed their clinical significance based on database annotations.

Furthermore, we annotated the disease phenotypes associated with both direct and adjacent mutations to identify potential functional consequences of altered phosphorylation dynamics. This integrated approach allowed us to explore not only the mutational effect on the autophosphorylation sites but also to uncover patterns that may suggest mechanistic links between potential orphan autophosphorylation sites associated with diseases.

### 4.7. Kinase Family-Based Conservation and Structural Mapping

To further characterize autophosphorylation sites, we performed kinase family-level conservation analysis using a scoring scheme adapted from our previous study [27]. The conservation score (0–1 scale) was computed based on sequence alignment features, and the criteria and formulation were directly adopted from the established methodology.

To provide structural context, we examined the spatial distribution of selected autophosphorylation sites with respect to key functional regions, including the protein kinase domain and activation loop, using AlphaFold-predicted structures for representative kinases.

### 4.8. Statistical Analysis

To analyse the significant coregulating phosphosite pairs, we employ *p*-values calculated by Fisher’s exact T test using the following formula:

$$\sum p=\;\frac{\left(a+b\right)!\left(c+d\right)!\left(a+c\right)!\left(b+d\right)!}{n!}\;\sum_{i}\frac{1}{a_{i}!b_{i}!c_{i}!d_{i}!}$$

We used a contingency table for FET, created using the following parameters:

*a* = n_00: Number of experimental conditions in which both sites were not detected.

*b* = n_U0 + n_D0 + n_0U + n_0D: Number of experimental conditions in which either of the sites was up-/downregulated while the other site was not detected.

*c* = n_UD + n_DU: Number of experiment conditions in which both sites show opposite co-regulation.

*d* = n_UU + n_DD: Number of experiment conditions in which both sites show identical co-regulation.

FET *p*-values were adjusted using multiple hypothesis testing (Benjamini–Hochberg false discovery rate (FDR) correction method). The resulting q-values represent the minimum false discovery rate. Phosphosite pairs with a FET *p*-value < 0.05 and q-value < 0.05 were considered significant.

## Figures and Tables

**Table 1:** (**A**): Positive co-regulation of orphan autophosphorylation sites with regulatory phosphosites (**B**): negative co-regulation of orphan autophosphorylation sites with regulatory phosphosites.

(**A**)					
**Regulatory Site**	**Regulatory Function**	**Orphan Autophosphorylation Site**	**n_uudd/n_uddu**	**FET *p*-Value**	**FDR**
MTOR_S2481	Activation	MTOR_S2478	68	1.96 × 10^−28^	1.43 × 10^−23^
EPHA2_Y594	Activation	EPHA2_Y575	30	2.02 × 10^−17^	1.61 × 10^−12^
RIPK2_S176	Activation	RIPK2_S178	12	7.46 × 10^−10^	5.17 × 10^−5^
RPS6KA1_S380	Activation	RPS6KA1_S45	7	1.76 × 10^−5^	1.45 × 10^−3^
RPS6KA1_S380	Activation	RPS6KA1_S732	7	5.13 × 10^−4^	1.99 × 10^−2^
(**B**)					
**Regulatory Site**	**Regulatory Function**	**Orphan Autophosphorylation Site**	**n_uddu/n_uudd**	**FET *p*-Value**	**FDR**
PRKAA1_S496	Inhibition	PRKAA1_S360	9	9.18 × 10^−3^	8.75 × 10^−4^
PRKAA1_S496	Inhibition	PRKAA1_S356	9	2.62 × 10^−4^	2.27× 10^−2^
PRKAA1_S496	Inhibition	PRKAA1_T355	8	5.06 × 10^−5^	1.20 × 10^−2^
PRKAA1_T183	Activation	PRKAA1_S360	8	1.35 × 10^−8^	4.96 × 10^−7^

SUM_UUDD: Total number of positively regulated experimental conditions; SUM_UDDU: total number of negatively regulated experimental conditions; n_uudd/n_uddu: ratio between SUM_UUDD and SUM_UDDU; n_uddu/n_uudd: ratio between SUM_UDDU and SUM_UUDD. FET *p*-value < 0.05; FDR < 0.05.

**Table 2:** Positive coregulation of orphan autophosphosites in kinases with known substrate sites.

Subatrate_Phosphosite	Orphan Autophosphorylation Site	UUDD/UDDU	Source	FET *p*-Value	FDR
NCK1_S85	PKN2_S583	44	PSPA_UUDD; Sugiyama_UUDD	2.41 × 10^−14^	1.88 × 10^−9^
RETREG3_S260	MARK2_S571	41	PSPA_UUDD	2.83 × 10^−22^	5.90 × 10^−18^
SFSWAP_S868	SRPK1_S309	41	PSPA_UUDD	3.40 × 10^−10^	4.55 × 10^−7^
SRRM2_S1014	CDK13_S358	40	PSPA_UUDD	1.19 × 10^−11^	4.48 × 10^−9^

n_uudd/n_uddu: Ratio between SUM_UUDD and SUM_UDDU.

**Table 3:** Co-detection frequency of orphan autophosphorylation site with substrate phosphorylation site.

Orphan Autophosphosite	Substrate Phosphosite	Substrate Site Function	Frequency	PMID_Count
STK10_S438	HSPB1_S82	Altered	831	150
STK10_S438	ALDOA_S39		669	112
RPS6KA3_S715	NUFIP2_S652		634	120
RPS6KA3_S715	ACLY_S455		623	119
AAK1_T606	STMN1_S16	Inhibition	620	108
RPS6KA3_S715	HSPB1_S82	Altered	620	117
RPS6KA3_S715	FXR2_S601		615	110
RPS6KA3_S715	SRRM2_S2692		611	110
RPS6KA3_S715	AHNAK_S135		610	118

Frequency: Number of experimental conditions in which sites were identified; PMID_count: number of articles in which sites were reported.

## Data Availability

The original contributions presented in this study are included in the article/Supplementary Materials. Further inquiries can be directed to the corresponding author.

## Supplementary Materials

The following supporting information can be downloaded at https://www.mdpi.com/article/10.3390/ijms27156745/s1.
