# Supplementary material for: Phosphoproteomics Data-Driven Integrative Analysis of Autophosphorylation Sites in the Human Kinome
Source: Int J Mol Sci. 2026 Jul 28;27(15):6745. doi: 10.3390/ijms27156745 (PMC13466655; doi:10.3390/ijms27156745)
Supplement: Supplementary file 1 [file ijms-27-06745-s001.zip › Supplementary Figure S1.pdf]

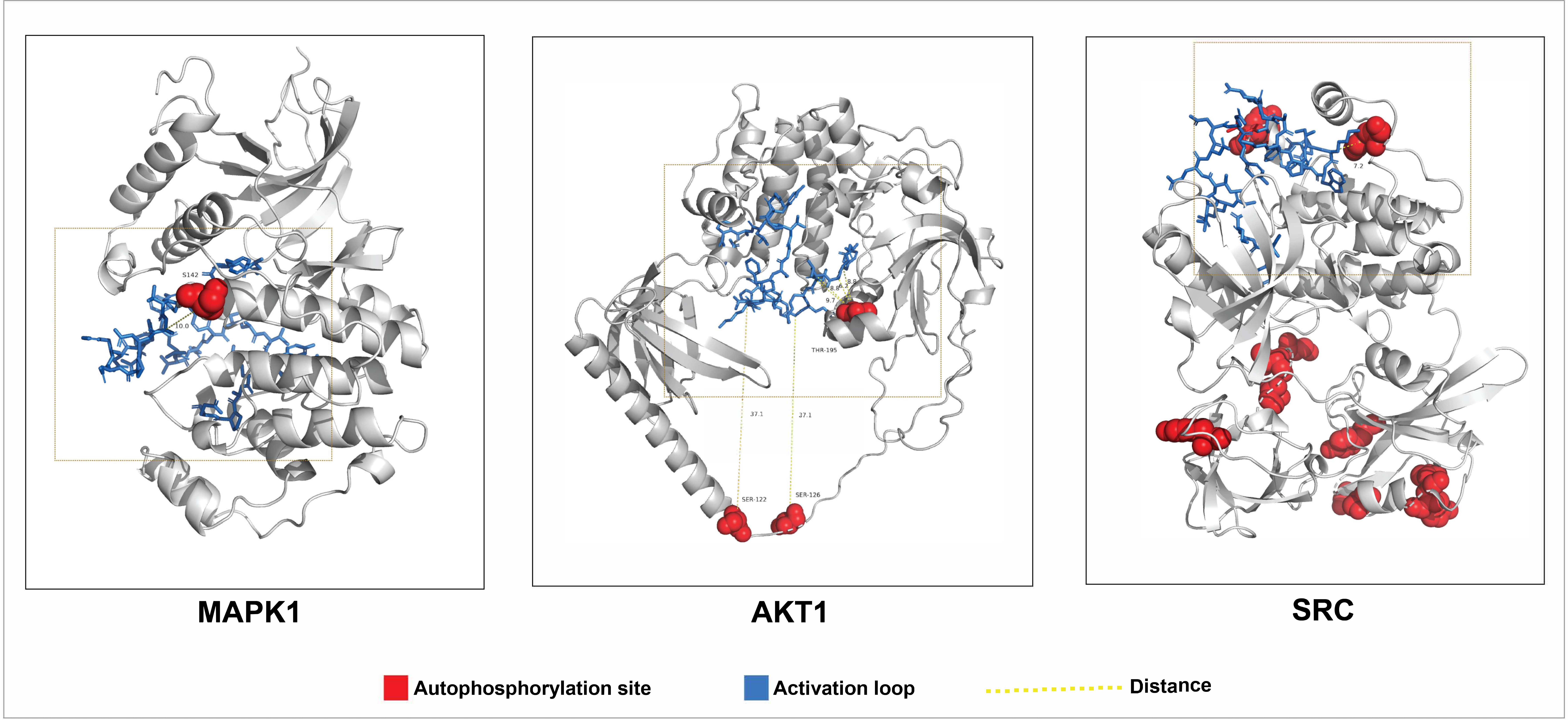

Supplementary Figure S1: Structural mapping of orphan autophosphorylation sites near to activation loop. Autophosphorylation sites mapped on the AlphaFold structures of kinases MAPK1, AKT1 and SRC. Phosphosites were represented as red color ball structures. Blue stick representations indicates the activation loop in that particular kinase. The distance between the autophosphorylation sites and the residues within the activation loop were represented in yellow dotted lines. The distance less than 5 Å represented in the box.
